# Influence of Polymer Fibers on the Structure and Properties of Modified Variatropic Vibrocentrifuged Concrete

**DOI:** 10.3390/polym16050642

**Published:** 2024-02-27

**Authors:** Evgenii M. Shcherban’, Sergey A. Stel’makh, Levon R. Mailyan, Alexey N. Beskopylny, Alla S. Smolyanichenko, Andrei Chernil’nik, Diana Elshaeva, Nikita Beskopylny

**Affiliations:** 1 Department of Engineering Geology, Bases, and Foundations, Don State Technical University, 344003 Rostov-on-Don, Russia; au-geen@mail.ru; 2 Department of Unique Buildings and Constructions Engineering, Don State Technical University, 344003 Rostov-on-Don, Russia; sergej.stelmax@mail.ru (S.A.S.); lrm@aaanet.ru (L.R.M.); chernila_a@mail.ru (A.C.); diana.elshaeva@yandex.ru (D.E.); 3 Department of Transport Systems, Faculty of Roads and Transport Systems, Don State Technical University, 344003 Rostov-on-Don, Russia; 4 Department of Water Supply and Sewerage, Don State Technical University, 344003 Rostov-on-Don, Russia; arpis-2006@mail.ru; 5 Department Hardware and Software Engineering, Don State Technical University, 344003 Rostov-on-Don, Russia; beskna@yandex.ru

**Keywords:** polymers, vibrocentrifuged concrete, variatropic concrete, concrete modification, fiber concrete, polymer fiber

## Abstract

The application of polymer materials in concrete structures is widespread and effectively used. However, there is a lack of a systematic knowledge base about the structure formation and properties of variatropic vibrocentrifuged modified fiber-reinforced concrete. The purpose of this work is the investigation of the influence of polypropylene (PF) and basalt fiber (BF) and modification with microsilica (MS) on the properties of variatropic concretes obtained using the synthesized vibration centrifugation technology. Test samples were made using vibration centrifugation technology, followed by sawing. Various types of fiber reinforcement were studied, both individually and in combination. To determine the degree of effectiveness of each recipe solution, the following main characteristics were monitored: the density and workability of concrete mixtures; the density of hardened composites; compressive strength (CS); bending strength (BS); water absorption (WA). In variatropic vibrocentrifuged concrete, the greatest efficiency is achieved with dispersed BF reinforcement in an amount of 1.5%. Compared to the control composition, the increase in CS was 8.50%, the increase in BS was 79.17%, and WA decreased by 27.54%. With PF reinforcement, the greatest effect was recorded at a dosage of 1.0%. The increase in CS was 3.16%, the increase in BS was 10.42%, and WA decreased by 17.39%. The MS modification showed the best effect with 8% replacement of part of the Portland cement. The increase in CS was 17.43%, the increase in BS was 14.58%, and WA decreased by 33.30%. The most effective and economically rational formulation solution for vibrocentrifuged concrete is combined fiber reinforcement in combination with the MS modification in the following quantities: BF—1.0%; PF—0.5%; MS—8%. The increase in CS was 22.82%, the increase in BS was 85.42%, and WA decreased by 37.68%.

## 1. Introduction

The use of polymer materials in construction is developing rapidly and is becoming an increasingly important aspect of progress in the construction industry. Various dispersed materials are no exception, used in many countries as reinforcing microfiber for building structures, and primarily for composite composition, in particular concrete [1,2,3]. As for concrete composites, preference in the most developed construction technologies is given to the most efficient structures in terms of mechanical, physical, energy and operational characteristics. In this regard, variatropic concretes are one of the most effective and progressive types of concrete [4,5,6].

It is known that concrete, as a broad concept, is often reinforced with fiber to improve mechanical performance characteristics, change the nature of the destruction of building structures based on such concrete and, in general, fiber-reinforced concrete is very popular in construction, and the regulatory and technical framework is being improved in accordance with this. Despite this, there are many scientific deficiencies and gaps in the regulatory and technical documents and in scientific research on the reinforcement of cement concrete with fiber. Such scientific deficiencies are especially pronounced in non-traditional types of fibers for normal-density concrete and, first of all, for variatropic concrete [7]. Note that in the technology of normal-density concrete, durable types of fibers, such as steel and basalt, are often used, and polypropylene polymer fibers are used much less frequently [8,9,10,11,12,13,14,15,16,17,18,19,20,21]. However, in our opinion, an interesting direction that can lead to a number of new results is dispersed reinforcement of variatropic concrete with polymer fibers, namely polypropylene fiber.

Vibrating centrifuged concrete (VCC) is a product made using centrifugal compaction technology using additional technological solutions that vibrate the mold with the concrete mixture during its compaction. Concrete made using this technology has a variatropic structure. A feature of concrete of this structure is its uneven density along the horizontal section [4,5]. The outer layer of the product consists mainly of coarse aggregate with a small amount of cement–sand mortar and is the strongest, the middle layer mainly consists of cement–sand mortar and smaller grains of coarse aggregate, and the inner layer includes the same mortar with less fine aggregate and a high water–cement ratio, since excess water is squeezed out of the upper and middle layers due to the action of centrifugal forces and moves to the inner layer [6,7]. The use of various prescription techniques for modifying VCC is a rather promising and poorly studied issue. Modification of variatropic concrete will strengthen and improve the structure of the entire composite, including the problematic inner layer, which has the lowest density, strength, and high porosity. The most popular type of modification of normal-density concrete is the application of various types of mineral additives and fibers [22,23].

One of the most popular and effective mineral additives used in the cement composites is microsilica [24]. By substituting 8% of cement with microsilica in the cited works [9,25], it was feasible to achieve concrete with heightened compressive strength and diminished permeability. In a study [23], including microsilica into the concrete composition, where part of the sand was replaced by crushed electronic plastic waste up to 20%, made it possible to minimize the negative impact of this waste on the strength characteristics of concrete and reduce its thermal conductivity. The presence of SiO_2_ in microsilica, comprising more than 95% of its composition, facilitates the formation of a secondary gel of calcium silicate hydrates (CSH) during the hydration reaction. This process contributes to the enhancement of concrete’s physical and mechanical properties, as well as its durability [24,25,26,27].

Polypropylene and basalt fibers are the most popular and most often used in the technology of normal-density concrete, and the effectiveness of their use has been confirmed by many scientific studies [8,11,12,13,14,15,16,17,18,19,20,21,28,29,30,31,32,33,34,35,36,37,38,39,40,41,42,43,44,45,46,47,48,49,50,51]. Basalt fiber is of inorganic origin, obtained by melting basalt rock in smelting furnaces and drawing continuous threads from this melt through special dies, which are subsequently dried and chopped into fibers. Basalt fiber has a fairly wide range of applications and is also used in the technology of asphalt concrete, geopolymer and brake friction composites [52]. Polypropylene fibers are synthetic formed from a melt of polypropylene. Polypropylene fiber is produced by extrusion. Special raw materials are brought to high temperatures and passed through holes with an individual profile and cross-section, and then the resulting fibers are cut to the required length [8,28,29,30,31,32,33,34].

In [9], the introduction of basalt fiber in an amount of 0.2% increases the crack resistance and bearing capacity of concrete beams. The use of basalt fiber in [12,13] during the manufacture of concrete-filled round steel tubular columns with recycled aggregate can improve their strength characteristics. In works [14,15,16,17], the introduction of basalt fibers in dosages from 0.5% to 1.5% is the most effective and improves such characteristics of cement composites as compressive strength, bending strength and impact strength. In addition to improving strength characteristics, the introduction of this type of fiber into cement composites also improves a number of physical characteristics, such as permeability, water absorption, frost resistance and thermal conductivity [18,19,20,21].

The introduction of polypropylene fibers into the composition of normal-density concrete also helps to improve its characteristics. For example, in works [8,31], concrete containing polypropylene fiber showed better frost resistance. During multiple freeze–thaw cycles in an aggressive environment, polypropylene fibers effectively suppress the propagation of microcracks and prevent concrete destruction. In a study [32], the authors were able to achieve a significant improvement in compressive and splitting strength, a decrease in water absorption and chloride ion permeability by introducing 0.3% polypropylene fiber. Polypropylene fiber in the composition of cement composites has a positive effect on their strength characteristics, namely on bending and splitting strength to a greater extent and to a lesser extent on compressive strength, also increases impact resistance and reduces permeability [33,34,35,36,37,38]. The optimal percentage of dispersed reinforcement with this type of fiber varies from 0.1% to 1.0% [39,40,41,42].

A popular trend is the simultaneous use of several types of fiber in a cement composite. As an illustration, in reference [43], the addition of basalt fiber at a concentration of 0.2% and polypropylene at a concentration of 0.1% to a cement hybrid mortar resulted in an 18.53% increase in compressive strength and a 21.73% increase in bending strength. By utilizing “a combination of basalt and polypropylene fibers in the scope of research” [44,45,46], it becomes feasible to enhance the resistance of cement composites against diverse forms of aggressive influences and enhance their durability attributes. The effectiveness, expressed in the increase in mechanical characteristics and impact resistance of cement composites dispersedly reinforced with a combination of basalt and polypropylene fibers in rationally selected dosages, is confirmed by a number of the following studies [47,48,49,50,51].

Considering the fact that more effective types of variatropic concretes have been previously established, specifically those obtained using vibration centrifugation technology, it will be interesting to study the structure, properties, and their mutual influence depending on the composition of vibrocentrifuged variatropic concretes based on polypropylene fiber. Given that we have previously established options for effective modification of vibrocentrifuged variatropic concretes with finely dispersed components [23,53,54], an even more interesting question is the study of polymer polypropylene fiber reinforcement of vibrocentrifuged variatropic concretes modified with various additives. Based on this, we formulate the purpose and objectives of this study. The purpose of the research is to develop effective compositions and study the influence of compositions, structure and properties on each other for vibrocentrifuged variatropic concretes modified with additives, reinforced with various types of fibers, and primarily polymer ones. The objectives of the research is to conduct large-scale experimental and theoretical studies to establish the effect of polymer polypropylene fiber on variatropic concretes of various compositions in combination with basalt fiber and microsilica, analyze the results obtained and develop new scientific provisions and proposals for the practical, production and construction industries.

## 2. Materials and Methods

### 2.1. Meterials

The subsequent raw materials were employed as constituents in the production of experimental samples:

- Portland cement CEM I 52.5N (PC);
- Crushed sandstone (CrS);
- Quartz sand (QS);
- Microsilica MK-85 (MS) (NLMK, Lipetsk, Russia);
- Plasticizer Poliplast PC (P) (Poliplast-YUG, Russia);
- Polypropylene fiber (PF) (Pascal, Dzerzhinsk, Russia);
- Basalt fiber (BF) (Pascal, Dzerzhinsk, Russia).

The characteristics of Portland cement, both physical and mechanical, are outlined in Table 1.

**Figure 1:**
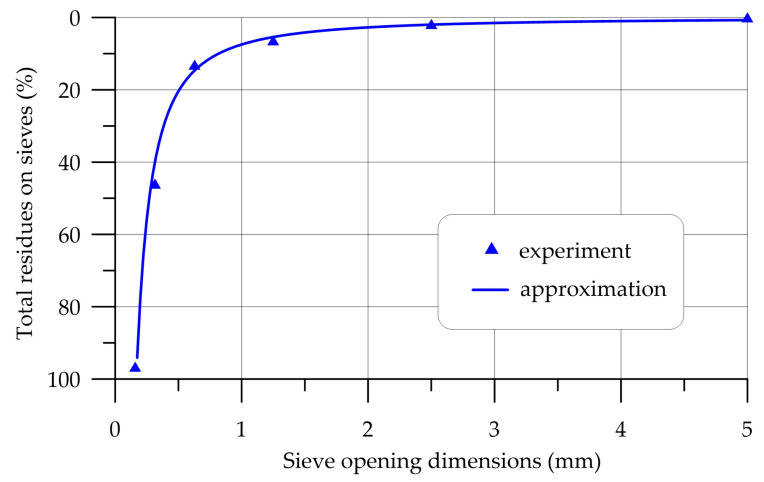
Particle size distribution curve of QS.

**Figure 2:**
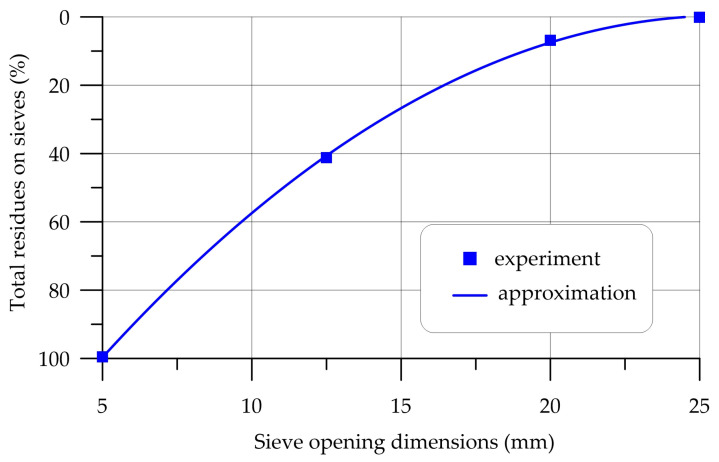
Particle size distribution curve of CrS.

The characteristics of coarse and fine aggregate are presented in Table 2 and Table 3 and Figure 1 and Figure 2.

The characteristics of the fiber are presented in Table 4.

MS characteristics are presented in Table 5.

**Figure 3:**
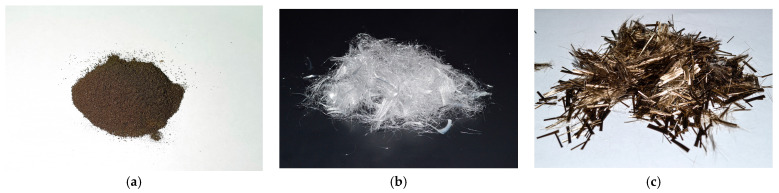
Modifier and fiber used in this study: (**a**) MS; (**b**) PF; (**c**) BF.

Table 5 data provided by the manufacturer. The appearance of the raw materials used to modify the VCC is presented in Figure 3.

### 2.2. Methods

The compositions of concrete mixtures for the manufacture of VCC samples were calculated, considering the requirements of [55] using the following method. At the beginning, the approximate required water–cement ratio was calculated using Formula (1):

(1) $$\frac{W}{PC}=\frac{0.45\;R_{PC}}{R_{b}+0.18R_{PC}}$$

Here, *W/PC* is a water–cement ratio; *R_PC_* is activity of cement (MPa); *R_b_* is the required strength of concrete (MPa) [56].

Then, the Portland cement content (kg/m^3^) was calculated:

(2) $$PC=\frac{W_{res}}{W/PC}$$

*W_res_* is residual water content in concrete after vibratory centrifugation, amounting to 150 L/m^3^.

Formulas (3)–(5) were utilized to determine the absolute volume of cement paste (*V_m_*) in liters, the absolute volume of the aggregate mixture (*V_A_*) in liters (QS + CrS), and the total weight consumption of aggregates (*A*) in kg, following the calculation of the required amount of cement:

(3) $$V_{m}=\frac{PC}{\rho_{PC}}+W_{m}$$

(4) $$V_{A}=1000\;-\;V_{m}$$

(5) $$A=V_{A}\times \rho_{A}$$

Here, $\rho_{PC}$ is the true density of Portland cement (kg/m^3^); *W_m_* is the approximate water consumption to obtain a concrete mixture with workability grade P1 (cone slump 1–4 cm) [56], which amounted to 190 l; $\rho_{A}$ is the reduced density of the sand-crushed stone mixture of, kg/m^3^ (expression (6)s):

(6) $$\rho_{A}=\frac{\rho_{CrS}+\rho_{QS}}{1+r}$$

$\rho_{CrS}$ is the true density of crushed stone (kg/m^3^); $\rho_{QS}$ is true sand density (kg/m^3^); *r* = 0.3.

The weight consumption of crushed stone and sand in kg was determined using Formulas (7) and (8):

(7) $$CrS=\frac{A}{1+r}$$

(8) QS=A−CrS

The calculated compositions of concrete mixtures for the production of vibrocentrifuged fiber-reinforced concrete, vibrocentrifuged concrete modified with the addition of microsilica, and vibrocentrifuged fiber-reinforced concrete modified with microsilica are presented in Table 6, Table 7 and Table 8.

**Figure 4:**
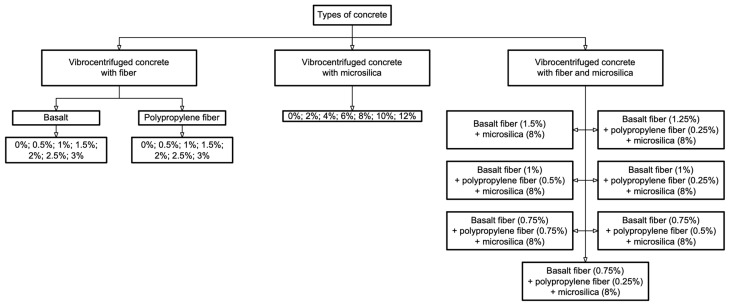
Experimental research program.

The experimental research program is presented in Figure 4.

The manufacturing process of vibrocentrifuged concrete samples was as follows.

**Figure 5:**
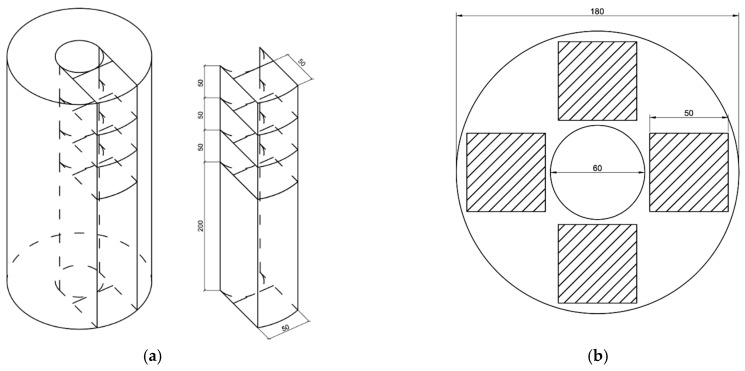
Scheme of sawing vibrocentrifuged elements (unit: mm): (**a**) isometric; (**b**) plan.

First, all raw materials for the concrete mixture were dosed in accordance with the recipe. PC, QS and additives (MS, PF, BF) were loaded into a laboratory concrete mixer BL-10 (ZZBO, Zlatoust, Russia) and mixed for 60 s. Subsequently, water containing a plasticizer was introduced to the dry mixture, followed by mixing the mixture until a uniform consistency was achieved for a duration of 120 s. During the final stage, CrS was added to the resulting mixture and all components were mixed for an additional 60 s. Next, the concrete mixture was poured into molds for centrifugation and installed on a laboratory installation for the production of ring-section concrete samples (DSTU, Rostov-on-Don, Russia) [57]. The samples were formed under the following centrifugation parameters (speed 150 rad/s; duration 12 min) and vibration parameters (height of clamp protrusions 5 mm, length of protrusions 20 mm, pitch between protrusions 30 mm). After molding, the sludge was drained, and the samples themselves were kept in the molds and removed from them a day later. After this, all samples of annular cross-section were kept in a standard hardening chamber KNT-1 (RNPO RusPribor, St. Petersburg, Russia) for 27 days at a temperature of 20 ± 2 °C and a relative humidity of 95%. According to the scheme presented in Figure 5, we performed the sawing of vibrocentrifuged specimens to determine the strength characteristics.

A total of 42 vibrocentrifuged elements with the following dimensions were manufactured and tested:

- Outer diameter D = 180 mm;
- Internal diameter d = 60 mm;
- Height h = 350 mm.

**Figure 6:**
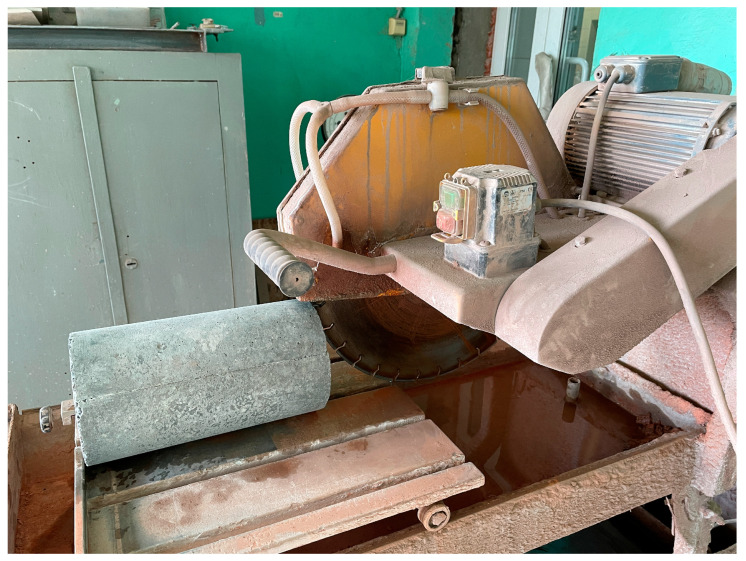
Process of sawing vibrocentrifuged elements.

**Figure 7:**
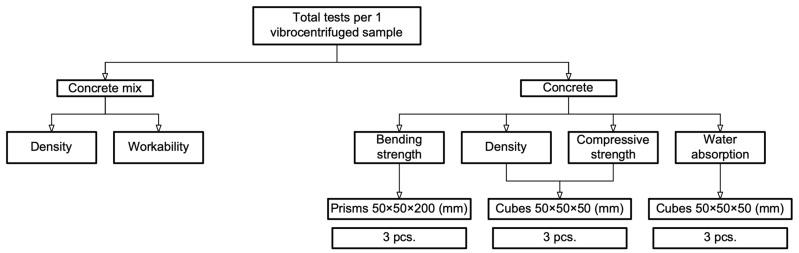
Test program for samples cut from vibrocentrifuged elements.

The process of sawing vibrocentrifuged elements on a stone-cutting machine (Helmut, Moscow, Russia) and the testing program for samples from them are presented in Figure 6 and Figure 7, respectively.

A total of 78 cube samples were tested for compression and 78 cube samples for water absorption, for a total of 156 samples. 78 prism samples were tested for bending. The total number of test samples was 234.

The determination of the average density and workability of concrete mixtures was carried out in accordance with the prescribed methods [58,59].

The method described in reference [60] was utilized to determine the density of hardened concrete.

The average density of concrete was calculated as the ratio of the mass of concrete to its total volume and was calculated using the formula:

(9) $$\rho =\frac{m}{V}\times 1000$$

Here, *m* is the mass of the sample (g); *V* is sample volume (cm^3^).

The compressive and bending strength of VCC samples were determined following the methodologies described in references [61,62,63,64], utilizing a Press P-50 installation from PKC ZIM in Armavir, Russia. During compression testing, cube samples were positioned on the lower support plate of the test press, with one of the chosen faces aligned centrally to its longitudinal axis, using marked indicators on the press plate. Following the installation of the sample onto the support plates of the press, it was subjected to a constant rate of load increase (0.6 ± 0.2) MPa/s until failure. The formula was employed to calculate the compressive strength:

(10) $$R=\alpha \frac{F}{A}$$

Here, *F* is the breaking load (N); *A* is the sample working section area (mm^2^); α is a scale factor for reducing the strength of concrete to the strength of concrete in samples of basic size and shape.

**Figure 8:**
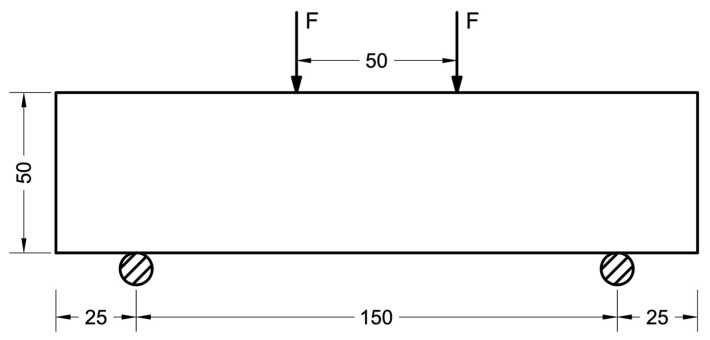
Bending test setup (unit: mm).

When testing for bending, the sample was installed on the press plate according to the scheme presented in Figure 8. Subsequently, the specimen underwent loading until reaching failure, with a constant rate of load increase of 0.05 ± 0.01 MPa/s.

The determination of flexural strength was conducted through the utilization of the following formula:

(11) $$R_{tb}=\alpha \frac{F\;l}{a\;b^{2}}$$

where *a*, *b*, *l* are the width, height of the cross-section of the prism and the distance between the supports, respectively, when testing samples for bending (mm).

The assessment of water absorption in concrete samples was conducted in compliance with the guidelines specified in regulatory documents [65,66]. In order to assess water absorption, concrete samples were submerged in a container filled with water, ensuring that the water level in the container exceeded the top level of the laid samples by approximately 50 mm. The saturated samples underwent weighing every 24 h, and the duration of the test extended until two consecutive weighings displayed a difference of no more than 0.1% in their results. The calculation of water absorption was performed using the following formula:

(12) $$W=\frac{m_{W}-m_{d}}{m_{d}}\times 100$$

where $m_{W}$ and $m_{d}$ are the mass of the water-saturated and dry sample (g).

The information presented on test standards implies the regulatory and technical basis of the proposed methods. However, it should be understood that regulatory technical documents and testing standards differ in different countries of the world and have certain nuances for each region. At the same time, it should be understood that the proposed variatropic vibrocentrifuged concrete has a number of important advantages over vibrated concrete and at the same time differs from it and has some complexity. Therefore, test standards represent an unconditional basis for determining the quality of concrete, but, nevertheless, it should be taken into account that with each new study in the direction of vibrocentrifuged concrete, experimental verification of the results obtained through their verification is necessary. It is recommended to use the standard methods of the country in which the research is being conducted as a baseline and repeat the results at least three times. This will eliminate random errors, the loss of certain results, and also assess the influence of all recipe and technological factors that create risks of not achieving a scientific result.

## 3. Results and Discussion

The results of determining the properties of a concrete mixture intended for the production of vibrocentrifuged fiber-reinforced concrete, vibrocentrifuged concrete modified with the addition of microsilica, and modified vibrocentrifuged fiber-reinforced concrete are presented in Table 9 and Table 10.

Based on the findings from the analysis of concrete’s fresh properties, as presented in Table 9 and Table 10, several dependencies can be observed. The utilization of fiber and microsilica within the investigated ranges does not yield any noteworthy influence on the modification of concrete mixture density. The density of concrete mixtures containing basalt fiber at dosages from 0.5% to 3.0% varied from 2233 kg/m^3^ to 2242 kg/m^3^, and with the same content of polypropylene fiber, it varied from 2235 kg/m^3^ to 2246 kg/m^3^. The density of concrete mixtures modified by adding microsilica in amounts from 2% to 12% varied from 2234 kg/m^3^ to 2245 kg/m^3^.

As for the characteristics of workability, expressed in cone slump, there is one common trend for both mixtures with fiber and mixtures with microsilica. With an increase in the content of fiber and microsilica, the workability of concrete mixtures decreases. For concrete mixtures containing basalt fiber in amounts of 0.5%, 1.0%, 1.5%, 2.0%, 2.5% and 3.0%, the reduction in cone settlement was 2.63%, 7.89%, 15.79%, 31.58%, 44.74% and 55.26%, respectively, in comparison with the control mixture composition. And for concrete mixtures containing 0.5%, 1.0%, 1.5%, 2.5% and 3.0% polypropylene fiber, the reduction in cone settlement was 5.26%, 7.89%, 21.05%, 28.95%, 47.37% and 63.16%, respectively. For concrete modified with the addition of microsilica in amounts of 2%, 4%, 6%, 8% and 10%, the reduction in cone settlement compared to the control composition was 10.53%, 18.42%, 28.95%, 34.21%, 47.37% and 55.26%, respectively. A decrease in cone settlement with an increase in fiber content can be explained by the friction that occurs between the surface of the fiber and the cement–sand mortar, which is also confirmed by the following studies [67,68]. Also, as the amount of fiber increases, the total surface area of the fibers also increases; accordingly, more cement paste is required to evenly lubricate all the fibers. As a result, with increasing fiber dosage, the concrete mixture will become more viscous and have a smaller cone slump [69]. When replacing part of the cement with a microsilica additive and increasing its dosage, the reduction in cone settlement is due to the fact that the presence of particles with a higher specific surface area compared to cement particles reduces the amount of freely bound water and increases the viscosity of the medium [70,71].

Next, Table 11 presents the results of determining the VCC density of all types of compounds manufactured in this study.

Based on the findings of determining the density of VCC, showed in Table 11, it can be concluded that the use of such formulation solutions as the introduction of fiber and microsilica to improve the physical and mechanical characteristics of VCC does not have a significant effect on the density of the hardened composite. With the introduction of basalt fiber from 0.5% to 3.0%, the density of concrete varies from 2389 kg/m^3^ to 2422 kg/m^3^. When adding the same amount of polypropylene fiber, the density changes from 2387 kg/m^3^ to 2413 kg/m^3^. For VCC modified with the addition of microsilica, the density varies from 2385 kg/m^3^ to 2411 kg/m^3^. Deviations in the density of composites of all developed types of compositions in comparison with the control sample, downward or upward, did not exceed 1%. This pattern is logical and justified. Firstly, fiber, both polypropylene and basalt, is introduced into concrete mixtures in small quantities from 0 to 3% by weight of cement and cannot significantly affect the final density of such a composite as concrete. The introduction of microsilica also does not have a significant effect on the final density of the composite due to its small amount from 0 to 12%.

**Figure 9:**
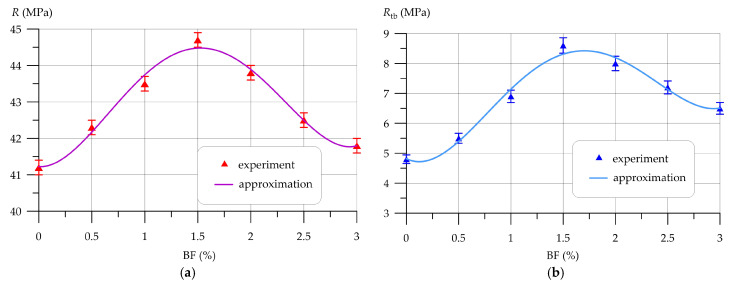
Dependence of VCC strength on basalt fiber content: (**a**) compression; (**b**) bend.

**Figure 10:**
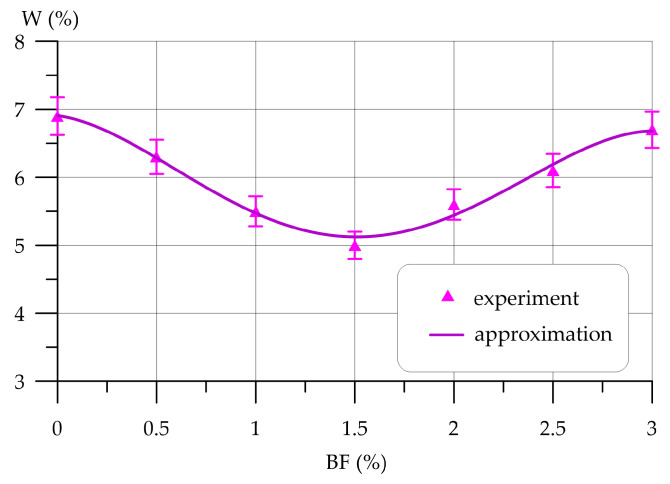
Change in water absorption VCC depending on BF content.

**Figure 11:**
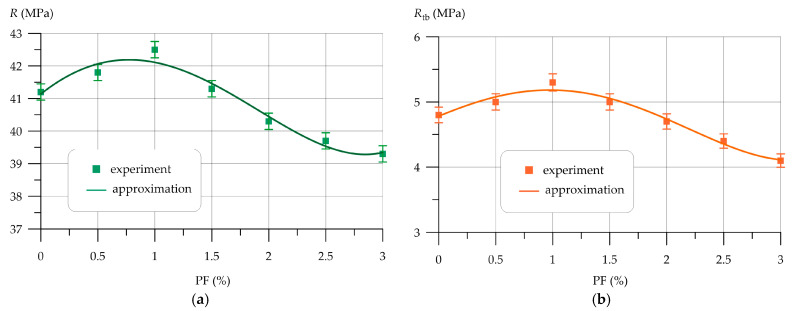
Variation in VCC strength as a function of PF content: (**a**) compression; (**b**) bend.

**Figure 12:**
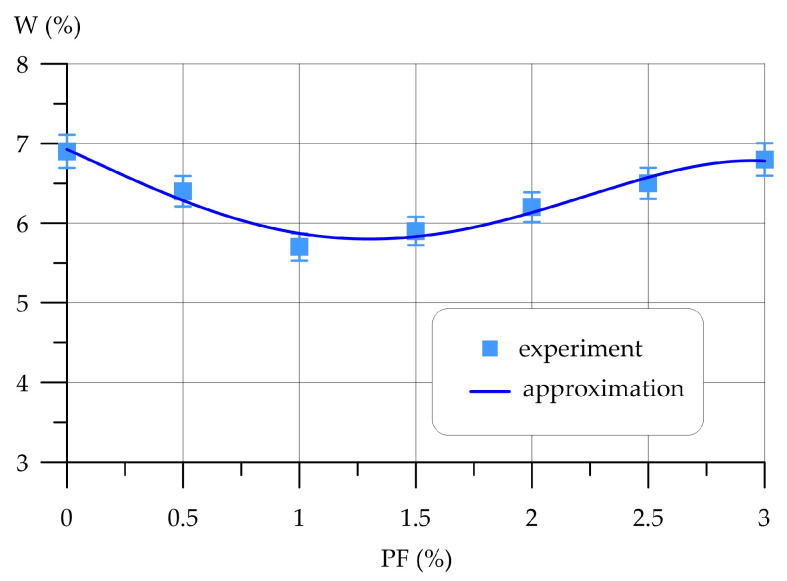
Variation in water absorption VCC depending on the amount of PF.

**Figure 13:**
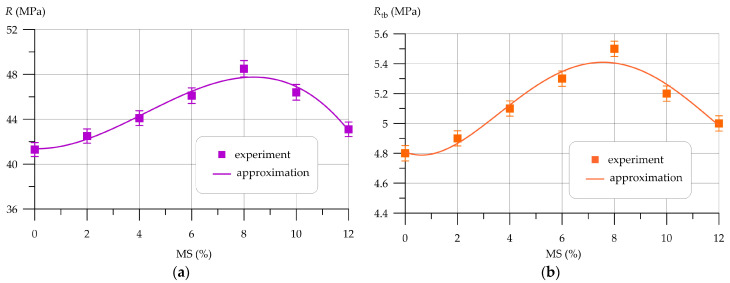
Variation in VCC strength as a function of the amount of MS: (**a**) compression; (**b**) bend.

**Figure 14:**
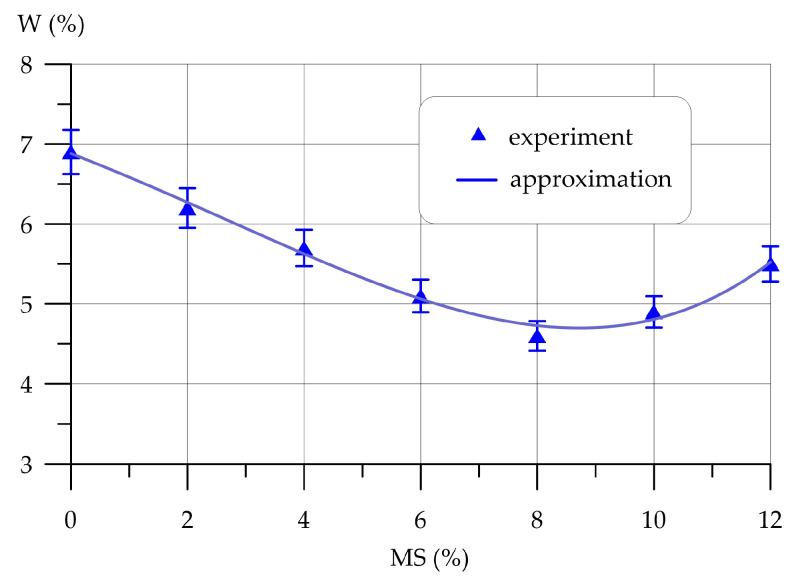
Variation in water absorption VCC depending on the amount of MS.

Subsequently, Figure 9, Figure 10, Figure 11, Figure 12, Figure 13 and Figure 14 depict the outcomes of the individual assessment of the physical and mechanical properties of VCC using basalt, polypropylene fiber, and microsilica.

The relationship between compressive and bending strength and the quantity of basalt fiber added to concrete is illustrated in Figure 9.

Dependences of VCC strength *R* and *R_tb_* on basalt fiber content BF (*x* in formulas) are approximated by a 4th-degree polynomial with coefficient of determination *R*^2^

(13) $$R=41.2-0.2148\;x+6.013\;x^{2}-3.941\;x^{3}+0.660\;x^{4},\;R^{2}=0.985$$

(14) $$R_{tb}=4.819\;-1.714\;x+7.765\;x^{2}-4.426\;x^{3}+0.697\;x^{4},\;R^{2}=0.981$$

The experimental findings, showcased in Figure 9, demonstrate a beneficial influence of incorporating basalt fiber in VCC technology on the concrete’s strength characteristics, as determined through compressive and flexural strength tests. The maximum values of compressive and bending strength were recorded at a BF content of 1.5%. The trends in changes in both compressive strength and flexural strength depending on the amount of fiber introduced into the concrete composition are similar. At 0.5% and 1.0% fiber, a smooth increase in strength is visible, then at a fiber amount of 1.5% a peak value is observed, and at BF dosages of 2.0%, 2.5% and 3.0% the strength characteristics begin to decrease. However, it is worth noting that VCC, dispersedly reinforced with BF in an amount of 0% to 3% by weight of PC, has higher compressive and flexural strength values compared to the control composition. Also, the introduction of BF has a much greater positive effect on the increase in bending strength. At BF contents of 0.5% and 1.0%, the increases in compressive strength were 2.67% and 5.58%, and the flexural strength increased by 14.58% and 43.75%, respectively. At 1.5% BF, the compressive strength increased by 8.5% and the flexural strength by 79.17%. Further, at 2.0%, 2.5% and 3.0% BF, the increases in compressive strength were 6.31%, 3.16% and 1.46%, respectively, and the flexural strength increased by 66.67%, 50.0% and 35.42%, respectively. Figure 10 graphically shows the change in water absorption of VCC containing different amounts of BF.

Dependence of water absorption *W* on BF content is approximated by a 4th-degree polynomial with coefficient of determination *R*^2^

(15) $$W=6.906-0.398\;x-2.498x^{2}+1.759\;x^{3}-0.296\;x^{4},\;R^{2}=0.982$$

From Figure 10, it can be seen that the introduction of BF in an amount from 0 to 3% has a positive effect on the water absorption of concrete, helping to reduce it. When adding 0.5% and 1.0% BF, a gradual decrease in water absorption is observed. Compared to the control composition, it decreased by 8.7% and 20.29%, respectively. The lowest water absorption value was recorded at a BF content of 1.5%. This dosage is more effective and reduces water absorption by 27.54%. With the addition of 2.0%, 2.5% and 3.0% BF, the effect of dispersed reinforcement with basalt fiber is reduced and water absorption is lower than the control composition by 18.84%, 11.59% and 2.90%, respectively.

As is known, the strengthening of concrete due to dispersed reinforcement is primarily due to the fact that the hardened concrete composite transfers the load to the fibers distributed in it due to tangential forces that act along the phase interface. Basalt fibers have a fairly high modulus of elasticity and absorb the bulk of the stresses, thereby increasing the overall strength of the composite [70]. Also, in addition to the properties of BF, an important point is the heterogeneity of the structure of the resulting VCC. The structural organization of fiber-reinforced concrete can be divided into two scale levels. The microscopic level of the structure of a concrete composite is determined by the phase composition of new formations and the nature of porosity. The macroscopic level is determined primarily by the type and properties of aggregates, hardened cement paste, fiber content and the uniformity of distribution of all these structural components among themselves [71]. At the macrostructure level, all components are interconnected by contacts, the strength of which will determine the basic properties of dispersed reinforced concrete. Thus, contacts between cement particles, between cement paste and filler, between cement paste, fiber and fillers will determine the final properties of the composite. The strength characteristics analysis revealed that the highest values of compressive and bending strength were achieved with a dosage of 1.5% basalt fiber. Accordingly, precisely at this percentage of dispersed reinforcement, VCC will have a dense and durable structure with the most uniform distribution of fiber and strong contacts of all components with each other. The lower values of compressive and flexural strength at 0.5% and 1.0% BF are due to the fact that the vibrocentrifuged composite is not fully saturated with BF. The decrease in characteristics with the introduction of 2.0–3.0% BF is associated with the supersaturation of the composite with fibers. When the composite is oversaturated with fibers, they cannot be evenly distributed and begin to tangle and form lumps. Lumps of fiber in the structure of the composite form places with weak strength, which subsequently negatively affects its properties [72,73]. The obtained increases in compressive strength and bending strength with the introduction of BF in a rational dosage are also comparable with the results obtained by other authors. For example, in studies [69,74,75,76,77], the introduction of basalt fiber into cement composites provided an increase in compressive strength of up to 10% and flexural strength of up to 65%.

The variation in compressive and flexural strength VCC due to different amounts of polypropylene fibers is shown in Figure 11.

Dependences of strength *R* and *R_tb_* as a function of PF content (*x* in formulas) are approximated by a 4th-degree polynomial with coefficient of determination *R*^2^

(16) $$R=41.13+2.775\;x-1.851\;x^{2}-0.0484\;x^{3}+0.0969\;x^{4},\;R^{2}=0.962$$

(17) $$R_{tb}=4.782\;+0.717\;x-0.156\;x^{2}-0.216\;x^{3}+0.0545\;x^{4},\;R^{2}=0.972$$

From the dependences of changes in strength characteristics presented in Figure 11, it was found that the introduction of polypropylene fiber into the VCC composition has a positive effect at dosages of 0.5% and 1.0% by weight of the binder. When introducing PF more than 1.0%, an effect is observed, expressed in a decrease in both compressive strength and bending strength. With a PF content of 0.5% and 1.0%, the increase in compressive strength was 1.46% and 3.16%, respectively, and the increase in flexural strength was 4.17% and 10.42%. At 1.5% PF, the compressive and flexural strengths were 0.24% and 4.17% higher compared to the control composition. At 2.0%, 2.5% and 3.0% PF, the compressive strength values decreased compared to the control composition by 2.18%, 3.64% and 4.61%, respectively. The reduction in flexural strength compared to the control composition was 2.08%, 8.33% and 14.58%, respectively.

The relationship between water absorption VCC and the quantity of polypropylene fiber added is illustrated in Figure 12.

Variation in water absorption VCC depending on the amount of PF is approximated by a 4th-degree polynomial with coefficient of determination *R*^2^

(18) $$W=6.927-1.335\;x-0.141\;x^{2}+0.535\;x^{3}-0.115\;x^{4},\;R^{2}=0.950$$

As can be seen from Figure 12, polypropylene fiber in an amount from 0% to 3% has a positive effect on the water absorption of VCC. The curve of changes in water absorption has three characteristic sections. At 0.5% PF, the decrease in water absorption compared to the control sample was 7.25%. At 1.0% PF, the maximum effect is observed, at which water absorption is reduced by 17.39%. At polymer fiber amounts of 1.5%, 2.0%, 2.5% and 3.0%, the water absorption VCC is less than the VCC of the control composition by 14.49%, 10.14%, 5.80% and 1.45%.

As is known, polypropylene fiber has a lower elastic modulus and tensile strength compared to basalt fiber [70]. Accordingly, the use of this fiber cannot significantly improve the strength properties due to the fact that concrete cannot transfer the forces generated in it to polypropylene fibers, which have lower strength [71]. According to the results obtained, it was established that polypropylene fiber has a positive effect on the strength characteristics of VCC when its content is up to 1% by weight of the binder. The introduction of fiber more than 1.0% negatively affects the compressive and flexural strength. This dependence of the behavior of PF as part of VCC can be explained as follows. The most uniform distribution of PF in the composite structure, achieved with its amount of 1.0%, provides a slight increase in strength characteristics due to the creation of macrostructural cells “coarse aggregate–fiber–cement–sand mortar”. Contacts between the main components of a given structural cell will provide an increase in compressive and bending strength. When the content of polypropylene fiber is more than 1.0%, its fibers cannot spread evenly, become tangled and form, as in the case of basalt fiber, lumps. At the same time, zones inside the composite body with a large and dense concentration of PF will have low strength, which negatively affects the overall final strength of the entire composite [72,78]. The positive effect of fiber on the properties of heavy concrete is also confirmed by the following studies [32,79,80,81]. For example, in [79], the introduction of PF improves the structure of the samples and improves the strength characteristics; however, when the structure of the composite is oversaturated with polypropylene fibers, a negative effect is observed. In a study [82], the introduction of polypropylene fiber into the composition of radiation-protective concrete with barite aggregates made it possible to increase the compressive strength to 19.8%. Similarly, in works [32,80,81], correctly selected dosages of polypropylene fiber improved the strength characteristics of composites and reduced their permeability.

Based on the test results of VCC dispersedly reinforced with various types of fibers—basalt and polypropylene—the following conclusions can be drawn:

- The introduction of fiber in an optimal dosage makes it possible to improve the physical and mechanical characteristics of VCC;
- Basalt fiber is more effective in comparison with polypropylene fiber; the increases in compressive and flexural strength VCC with 1.5% BF were 8.5% and 79.17%, respectively, and water absorption decreased by 27.54%; the increases in compressive and flexural strength at 1.0% PF were 3.16% and 10.42%, respectively, and water absorption decreased by 17.39%.

Figure 13 and Figure 14 show the results of determining the physical and mechanical characteristics of VCC modified by MS.

Variations in VCC strength *R* and *R_tb_* as a function of the amount of MS (*x* in formulas) are approximated by a 4th-degree polynomial with coefficient of determination *R*^2^

(19) $$R=41.37-0.0651\;x+0.290\;x^{2}-0.0231\;x^{3}+2.367\;x^{4},\;R^{2}=0.970$$

(20) $$R_{tb}=4.809-0.0728\;x+0.0656\;x^{2}-0.00798\;x^{3}+0.00026\;x^{4},\;R^{2}=0.950$$

The introduction of MS into VCC improves the strength properties. The curves of changes in compressive and bending strength depending on the amount of MS have three characteristic sections. When MS is introduced in an amount of 2–6%, a uniform increase in compressive and flexural strength is observed from 2.91% to 11.62% for compressive strength and from 2.08% to 10.42% for flexural strength. At 8% MS content, the maximum increase in compressive strength by 17.43% and flexural strength by 14.58% is observed, which indicates the greatest effectiveness of MS introduced into VCC in this amount. When MS is more than 10%, its efficiency decreases, and the increase in compressive and bending strength at MS 10% and 12% was 12.35% and 4.36%, and 8.33% and 4.17%, respectively.

Variation in water absorption VCC depending on the amount of MS is approximated by a 4th-degree polynomial with coefficient of determination *R*^2^

(21) $$W=6.881-0.283\;x-0.0138\;x^{2}+0.001199\;x^{3}+9.46\times 10^{-5}\;x^{4},\;R^{2}=0.990$$

From Figure 14, it can be seen that the introduction of MS has a positive effect on water absorption and helps to reduce it. The curve of changes in water absorption also has three characteristic sections. A smooth decrease in water absorption is observed when the amount of MS is from 2% to 6%. The peak value of the minimum percentage of water absorption was recorded for samples with 8% MS. However, when MS is introduced above 8%, its effectiveness begins to decrease and water absorption increases. For example, the water absorption of VCC modified by adding microsilica in amounts of 2%, 4%, 6%, 8%, 10% and 12% decreased by 10.1%, 17.4%, 26.1%, 33.3%, 29.0% and 20.3%, respectively. in comparison with the control composition.

The improvement in physical and mechanical properties of MS-modified VCC is attributed to the interaction mechanism of MS with PC and its distribution in the concrete composite. At an optimal MS content of 2% to 8%, microsilica particles having pozzolanic activity enter into a hydration reaction and contribute to the formation of additional calcium hydrosilicates, which strengthen the future structure of the composite [25,83]. MS particles also function as a mineral filler. They increase packing density by filling voids in the cement matrix. Accordingly, the structure of the vibrocentrifuged composite becomes denser and with fewer capillary pores. This mechanism of action of the microsilica additive and its effectiveness in concrete technology is also confirmed by the following studies [9,84,85].

Thus, through the use of various prescription techniques for VCC, rational dosages of each additive were established:

- The content of basalt fiber was 1.5% by weight of cement;
- The content of polypropylene fiber was 1.0% by weight of cement;
- The microsilica content was 8% instead of part of the cement by weight.

The use of basalt fiber in VCC technology with a rationally selected dosage makes it possible to increase the strength and physical characteristics of the material. At 1.5% fiber content, compressive strength increases by 8.5%, flexural strength by 79.17%, and water absorption decreases by 27.54%. Compared to basalt fiber, polypropylene fiber is less effective. The increase in compressive strength at 1.0% PF was 3.16%, flexural strength was 10.42%, and water absorption decreased by 17.39%. Compared to basalt fiber, polypropylene fiber has a lower elastic modulus, lower tensile strength and lower adhesion to other components of the concrete mixture. We also note that basalt and polypropylene fibers perform better under the action of bending loads on VCC [69,74,75,76]. If the use of fiber in the VCC composition helps to improve the physical and mechanical characteristics by improving the structure of the composite and creating strong macrostructural cells throughout its entire volume, then the use of microsilica improves the characteristics of concrete due to the interaction of microsilica particles with the components of the concrete mixture at the micro level [84].

**Figure 15:**
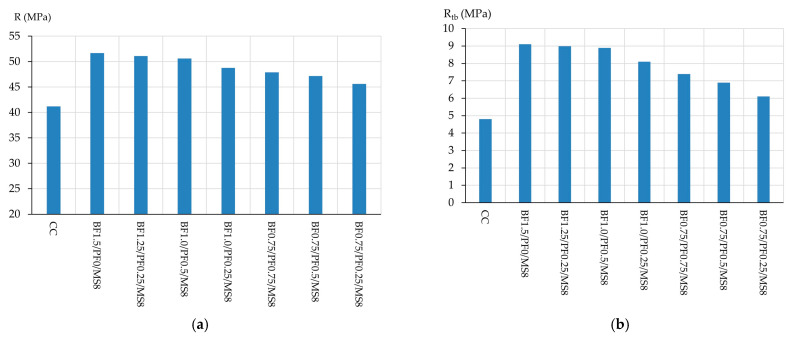
Change in the strength of modified vibrocentrifuged fiber-reinforced concrete: (**a**) compression; (**b**) bend.

**Figure 16:**
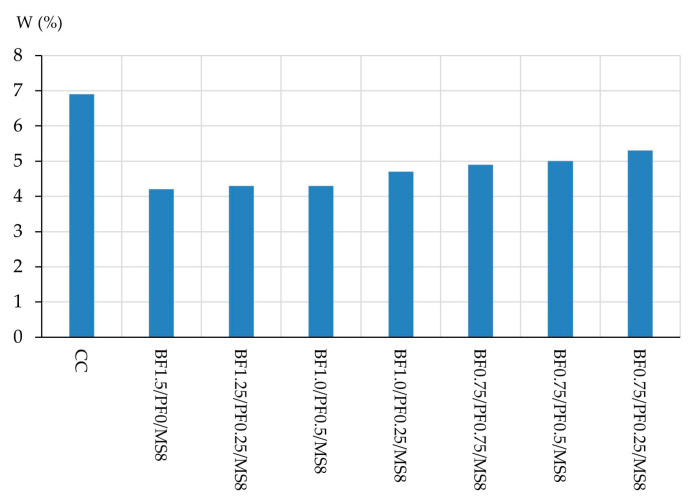
Change in water absorption of modified vibrocentrifuged fiber-reinforced concrete.

According to the data obtained above, the maximum percentage of combined fiber reinforcement, taking into account the types of fibers used, should not exceed 1.5%. Thus, the following quantities of each type of fiber for combined reinforcement were established, and the results of determining the fresh properties of concrete mixtures and the physical and mechanical characteristics of the modified vibrocentrifuged fiber-reinforced concrete are presented in Table 12 and Figure 15 and Figure 16.

The use of combined recipe solutions in VCC technology, as is the case with the use of each recipe solution separately, does not have a significant effect on the change in the density of concrete mixtures. Regarding the workability of concrete mixtures of compositions such as BF1.5/PF0/MS8, BF1.25/PF0.25/MS8, BF1.0/PF0.5/MS8, BF1.0/PF0.25/MS8, BF0.75/PF0.75/MS8, BF0.75/PF0.5/MS8, BF0.75/PF0.25/MS8, then it is lower in comparison with the workability of the control composition by 28.95%, 34.21%, 31.58%, 28.95%, 36.84%, 23.68% and 21.05%, respectively.

As can be seen from Figure 15a, the highest value of compressive strength was obtained for composition type BF1.5/PF0/M8, which is logical and comparable with the test results described above, and the increase in compressive strength itself was 25.49% in comparison with control composition. Compositions such as BF1.25/PF0.25/MS8 and BF1.0/PF0.5/MS8 have slightly lower compressive strength values compared to the most effective VCC composition with BF and MS. The increases in compressive strength were 24.03% and 22.82%, respectively. As for compositions like BF1.0/PF0.25/MS8, BF0.75/PF0.75/MS8, BF0.75/PF0.5/MS8, BF0.75/PF0.25/MS8, a negative trend is already observed here. The effectiveness of these compositions, expressed in increases in compressive strength compared to the control, was 18.45%, 16.26%, 14.56% and 10.68%, respectively. In general, the decrease in the effectiveness of these combined reinforcement options can be explained by an increase in the content of polypropylene fiber. The flexural strength trend shown in Figure 15b is similar to the compressive strength trend. The highest flexural strength value is for composition type BF1.5/PF0/MS8, and the strength increase value was 89.58%. Compositions of type BF1.25/PF0.25/MS8 and BF1.0/PF0.5/MS8 showed an increase in flexural strength of 87.50% and 85.42%, respectively. As for compositions like BF1.0/PF0.25/MS8, BF0.75/PF0.75/MS8, BF0.75/PF0.5/MS8, BF0.75/PF0.25/MS8, they have lower efficiency, and the increase in flexural strength was 68.75%, 54.17%, 43.75% and 27.08%, respectively, compared to the control composition.

According to the dependence of the change in water absorption of modified vibrocentrifuged fiber-reinforced concrete with various options for combined reinforcement, presented in Figure 16, it is clear that the composition of type BF1.5/PF0/MS8 is the most effective and has the lowest water absorption value, which is 39.13% lower than the control one. Compositions BF1.25/PF0.25/MS8 and BF1.0/PF0.5/MS8 are also effective and have low water absorption values, which are 37.68% lower than the control. And the compositions BF1.0/PF0.25/MS8, BF0.75/PF0.75/MS8, BF0.75/PF0.5/MS8, BF0.75/PF0.25/MS8, as in the case of strength characteristics, are less effective. The values of reduction in water absorption for these compositions in percentage terms, in comparison with the control, were 31.88%, 28.99%, 27.54% and 23.19%.

Based on the results of determining the characteristics of modified vibrocentrifuged fiber-reinforced concrete with different proportions of fibers, it can be concluded that compositions of three types BF1.5/PF0/MS8, BF1.25/PF0.25/MS8 and BF1.0/PF0.5/MS8 are the most effective, and the values of their physical and mechanical characteristics differ slightly. For example, the compressive strength of the most effective composition modified with 1.5% BF and 8% MS is only 2.17% higher compared to the composition modified with 1.0% BF, 0.5% PF and 8% MS. The difference in bending strength and water absorption for these compositions is 2.25% and 2.33%. Accordingly, in practice, in the manufacture of VCC, the most effective and efficient would be the use of a combined modification of the BF1.0/PF0.5/MS8 type. This is primarily due to the economic benefits of this formulation solution, since basalt fiber has a higher cost than polypropylene fiber. The lower efficiency of the combined modification options such as BF1.0/PF0.25/MS8, BF0.75/PF0.75/MS8, BF0.75/PF0.5/MS8, BF0.75/PF0.25/MS8 is primarily due to a decrease in the share of basalt fiber and an increase in the share of polypropylene fiber. Basalt fiber itself has a higher modulus of elasticity and tensile strength than polypropylene. Accordingly, when stresses arise in the structure of a vibrocentrifuged composite, these fibers absorb part of the stresses and prevent microcracks from forming in the structure of the composite. Also, basalt fiber has better adhesion to cement–sand mortar than polypropylene fiber [11,12,13,14,77].

In general, the combination of polypropylene and basalt fiber in a rational ratio allows one to achieve positive effects due to polyreinforcement. Polyreinforcement is a combination of two or more types of fiber with different geometric dimensions and elastic-plastic characteristics [70,71]. Theoretically, the structure of poly-reinforced concrete can be described as follows. The polypropylene and basalt fibers used in this study have different geometric parameters, which ensures the formation of macrostructural cells at different levels of the concrete structure. Smaller fibers are located in structural cells formed by larger fibers. With such a composite structure, smaller fibers are in the contact zone with larger fibers and prevent the formation of cracks at the crack formation stage and soften stresses [72]. However, this approach to explaining the effect of polyreinforcement is too idealized. A more accurate idea would be about the structure of fiber-reinforced concrete, where at each structural level two main phases can be distinguished: dispersed phase—fiber; dispersed medium is the body of the composite, in which all the main changes occur in the process of its formation. And the interactions of these phases with each other through the contact zone will determine the future characteristics of the composite. The effectiveness of the combined use of basalt and polypropylene fiber is also confirmed by a number of the following works [10,44,46,86,87]. The effect of polyreinforcement using polymer fiber and the introduction of microsilica additive has a complex effect on the entire structure of the vibrocentrifuged composite at the macro and micro levels. This makes it possible to obtain variatropic concrete with improved compressive strength, flexural strength, and reduced water absorption [52,88,89].

The results obtained are interesting from the point of view of fundamental science and applied problems and require detailed discussion. When discussing the results obtained, a comparison should be made between different formulation solutions and an assessment of each factor individually and in combination. As for individual factors, as mentioned earlier, the VCC modified with polypropylene fiber showed the least improvement in performance. And first of all, this is due to the fact that polypropylene fiber itself has lower strength and deformation characteristics than concrete itself, and cannot properly perceive the stresses arising in concrete and redistribute them [8,31,32]. Modification of VCC with basalt fiber is more effective, since basalt fiber has a higher elastic modulus, tensile strength, and when stresses occur in the composite structure, it prevents microcrack formation [11,12,13,14,69]. Modification with microsilica improves physical and mechanical characteristics due to processes occurring at the microlevel, promotes the formation of additional calcium hydrosilicates and reduces the capillary porosity of the composite [28,29]. At the same time, the best recipe and technological solution turned out to be combined reinforcement with modification in combination with the synthesized vibration centrifugation technology. This technology, in combination with modification and combined reinforcement (microsilica (8%) + basalt fiber (1.0%) + polypropylene fiber (0.5%)) made it possible to achieve the most dense packing of particles in the concrete structure at the microlevel and uniform distribution of fiber fibers in VCC with the formation of strong contacts at the interface between the phases “coarse aggregate–fiber–cement–sand mortar”. The structure formation of such concrete is regulated taking into account two factors: control of this structure and the formation of the properties of the conglomerate through a complex recipe and technological solution in the form of modification, combined reinforcement and increasing the degree of variatropy with the creation of a perfect structure due to the synthesized vibration centrifugation technology [5,90,91]. The resulting complex effect contributes to the best combination of structure and properties of combined-modified vibrocentrifuged variatropic concretes [5,7,22,23].

The research carried out is new, and in different directions. The first direction should reflect the research novelty. The novelty of this study lies, firstly, in the novelty of the proposed methodology. The research methodology involves a comprehensive study of a completely new technology for producing materials in combination with new approaches to reinforcing known materials with fiber. That is, the research simultaneously involves the study of technology, the study of recipes and the study of the processes of creating a new material. At the same time, the approach to studying the properties of a new material is integrally connected with the study of its structure. We put forward and successfully confirm a hypothesis about the perfection of a new type of structure, a new material obtained using a new technology using a new approach to fiber reinforcement. Research methods also, although based on standard methods, nevertheless make a number of reservations about the need for verification tests until the corresponding vibrocentrifuged technology for producing polymer-containing fiber-reinforced new variatropic concrete with an improved structure is included in the relevant standards and regulatory technical documents.

The resulting new technology differs from previously known ones, firstly, due to the additional vibration effect on centrifugally compacted concrete mixtures. The resulting material has a fundamentally new type of structure, an improved variatropic one, which assumes significant differences in the layers of new concrete of a reinforced concrete element having a ring cross-section, with differentiation of characteristics along this section. In this case, the polymer fiber has the character of a connecting link that allows connecting conventional layers of a differentiated cross-section and allows simultaneously achieving useful differentiation with a controlled type. This differentiation does not go beyond the scope of technological control and allows it to be used to the fullest, using all hidden strength and deformation reserves. The presented type of variatropic fiber-reinforced concrete structures with differentiation of properties along the cross-section also makes it possible to obtain an additional damping phenomenon when exposed to long-term and short-term loads with changes in the modulus of elasticity and deformability for use in difficult conditions, for example, in conditions of increased vibration industrial impacts of manufacturing enterprises, or in dynamic conditions transport loads, or in earthquake-prone areas, which in a certain way brings significant novelty to previously known centrifugally compacted reinforced concrete variatropic products and structures.

All of the above together makes a fundamentally new technology with the resulting fundamentally new material for fundamentally new types of structures an effective and useful direction for scientific research, construction production and the creation of new types of buildings and structures with increased performance characteristics, increased reliability and increased safety.

This creates the competitiveness of the manufactured product, namely vibrocentrifuged variatropic concrete reinforced with polymer fiber, confirms the competitiveness of the new technology, which, without a significant increase in labor, material costs and energy resources, makes it possible to obtain improved, advanced designs for buildings and structures operated absolutely anywhere in the world with high degree of efficiency, and such designs are designed to supplant more traditional and less efficient, more massive and less reliable designs made without improved structures and the use of the most useful and effective polypropylene fiber.

On a global scale, research is necessary to improve technologies for the manufacture of building products and structures, simplify construction technologies for the construction of buildings and structures, as well as promote the wider use of innovative polymer materials in new building structures. This is confirmed by surveys conducted among scientists, industrialists, and authorities of various states in all countries of the world.

Indeed, the transition to polymer building materials has a global scale, since modern construction involves the use of metal reinforcement and rods, which, firstly, increase the weight of the structure, and secondly, create conditions for increasing the cost of construction processes. All this is a problem for modern technologies, and therefore polymeric materials, including fiber, are a challenge on a global scale for builders and the production of building materials, as well as for research in this area.

## 4. Conclusions

An analysis was conducted on different formulation solutions to determine their effects on the fresh, physical, and mechanical properties of vibrocentrifuged concrete. The findings of this study can be succinctly summarized as follows.

(1) The addition of fiber and silica fume in all quantities considered does not have any significant effect on the density of vibrocentrifuged concrete. However, the use of these recipe solutions reduces the workability of concrete mixtures. The decrease in workability is direct in nature, that is, with an increase in the amount of fiber and microsilica content, the cone settlement decreases. In the case of fiber reinforcement, the decrease in cone settlement is explained by the fact that friction forces arise between the surface of the fiber and the cement–sand mortar, and part of the cement paste is used to lubricate the introduced fibers. These factors increase the viscosity of the concrete mixture. In the case of microsilica, the reduction in cone settlement is justified by the introduction of particles with a high specific surface area, the wetting of which requires more freely bound water.
(2) In vibrocentrifuged concrete, the greatest efficiency is achieved with dispersed reinforcement with basalt fiber in an amount of 1.5%. Compared to the control composition, the increase in compressive strength was 8.50%, flexural strength was 79.17%, and water absorption decreased by 27.54%.
(3) When modified with polypropylene fiber, the greatest effect was recorded at a dosage of 1.0%. The increase in compressive strength was 3.16%, flexural strength was 10.42%, and water absorption decreased by 17.39%.
(4) Modification with microsilica showed the best effect with 8% replacement of part of the cement. The increase in compressive strength was 17.43%, flexural strength was 14.58%, and water absorption decreased by 33.30%.
(5) The most effective and economically rational formulation solution for vibrocentrifuged concrete is combined fiber reinforcement in combination with modification with microsilica in the following quantities: basalt fiber—1.0%; polypropylene fiber—0.5%; microsilica—8%. The increase in compressive strength was 22.82%, flexural strength 85.42%, and water absorption decreased by 37.68%.
(6) The effect of combined reinforcement using polymer fiber and modification with microsilica has a complex effect on the structure and properties of the vibrocentrifuged composite and makes it possible to obtain variatropic concrete with improved compressive strength, flexural strength, and reduced water absorption.
(7) The prospects for the practical application and implementation of the results obtained are in the sphere of interest of manufacturers of concrete and reinforced concrete products, since such proposals can lead to a reduction in the percentage of defects in production and the production of high-quality products without a significant change in costs. Construction organizations will be stakeholders in this case, since they will receive safer and more reliable building products and structures that will be more resistant to various impacts, and due to the more viscous nature of destruction, will be more likely to allow the operation of buildings and structures erected in complex conditions. Prospects for the development of this research lie in the direction of testing other types of fibers, including polymer fibers made from other materials.
(8) The results obtained are pointwise for specific cases. The use of polymer fiber in vibrocentrifuged concrete, like any scientific or industrial discovery or innovation, has its limitations. Limitations on the applicability of the proposed scientific developments lie in the need for additional checks if they go beyond the presented areas. In particular, the proposed formulations are applicable for specific variatropic vibrocentrifuged products and structures, and the dosage of fiber, if it is replaced from polymer to other types, also needs to be clarified. Therefore, the specific accuracy of the results obtained in the studies performed should be taken into account.

## Figures and Tables

**Table 1:** Physical and mechanical characteristics of PC.

Property	Value
Specific surface area (m^2^/kg)	335
Soundness (mm)	0.3
Fineness, passage through a sieve No 008 (%)	97.3
Setting times (min) - start - end	190 270
Compressive strength (MPa): - 2 days - 28 days	26.2 56.7

**Table 2:** QS characteristics.

Property	Value
Bulk density (kg/m^3^)	1338
Apparent density (kg/m^3^)	2631
The content of dust and clay particles (%)	0.06
Content of clay in lumps (%)	0.09
Organic and contaminant content (%)	No
Fineness modulus (unit-less)	1.64

**Table 3:** Characteristics of CrS.

Property	Value
Bulk density (kg/m^3^)	1409
Apparent density (kg/m^3^)	2653
Resistance to fragmentation (wt %)	11.3
The content of lamellar and acicular grains (wt %)	8.5

**Table 4:** Characteristics of PF and BF.

Type of Fiber	Length, mm	Density, g/cm^3^	Tensile Strength, MPa	Modulus of Elasticity, GPa
PF	12–16	0.89	170	28
BF	12–24	2.6	1100	76

**Table 5:** MS characteristics.

Property	Value
Mass fraction of water (%)	0.15
Mass fraction of loss on ignition (%)	0.80
Bulk density (kg/m^3^)	252
SiO_2_ (%)	93
Al_2_O_3_ (%)	1.68
Fe_2_O_3_ (%)	0.65
CaO (%)	2.17
MgO (%)	1.01
K_2_O (%)	1.23
S (%)	0.26

**Table 6:** Designs of vibrocentrifuged fiber-reinforced concrete mixtures.

Mixture Type	Concrete Mixture Proportion Per 1 m^3^						
	PC (kg/m^3^)	W (L/m^3^)	CrS (kg/m^3^)	QS (kg/m^3^)	BF (kg/m^3^)	PF (kg/m^3^)	P (kg/m^3^)
Control composition (CC)	394	190	1287	546	0.0	-	3.9
BF0.5	394	190	1287	546	2.0	-	3.9
BF1.0	394	190	1287	546	3.9	-	3.9
BF1.5	394	190	1287	546	5.9	-	3.9
BF2.0	394	190	1287	546	7.9	-	3.9
BF2.5	394	190	1287	546	9.9	-	3.9
BF3.0	394	190	1287	546	11.8	-	3.9
PF0.5	394	190	1287	546	-	2.0	3.9
PF1.0	394	190	1287	546	-	3.9	3.9
PF1.5	394	190	1287	546	-	5.9	3.9
PF2.0	394	190	1287	546	-	7.9	3.9
PF2.5	394	190	1287	546	-	9.9	3.9
PF3.0	394	190	1287	546	-	11.8	3.9

**Table 7:** Designs of vibrocentrifuged concrete mixtures modified with the addition of microsilica.

Mixture Type	Concrete Mixture Proportion Per 1 m^3^					
	PC (kg/m^3^)	W (L/m^3^)	CrS (kg/m^3^)	QS (kg/m^3^)	MS (kg/m^3^)	P (kg/m^3^)
MS2	386.1	190	1287	546	7.9	3.9
MS4	378.2	190	1287	546	15.8	3.9
MS6	370.4	190	1287	546	23.6	3.9
MS8	362.5	190	1287	546	31.5	3.9
MS10	354.6	190	1287	546	39.4	3.9
MS12	346.7	190	1287	546	47.3	3.9

**Table 8:** Designs of silica fume-modified vibrocentrifuged fiber-reinforced concrete.

Mixture Type	Concrete Mixture Proportion per 1 m^3^							
	PC (kg/m^3^)	W (L/m^3^)	CrS (kg/m^3^)	QS (kg/m^3^)	BF (kg/m^3^)	PF (kg/m^3^)	MS (kg/m^3^)	P (kg/m^3^)
BF1.5/PF0/MS8	362.5	190	1287	546	5.4	-	31.5	3.9
BF1.25/PF0.25/MS8	362.5	190	1287	546	4.5	0.9	31.5	3.9
BF1.0/PF0.5/MS8	362.5	190	1287	546	3.6	1.8	31.5	3.9
BF1.0/PF0.25/MS8	362.5	190	1287	546	3.6	0.9	31.5	3.9
BF0.75/PF0.75/MS8	362.5	190	1287	546	2.7	2.7	31.5	3.9
BF0.75/PF0.5/MS8	362.5	190	1287	546	2.7	1.9	31.5	3.9
BF0.75/PF0.25/MS8	362.5	190	1287	546	2.7	0.9	31.5	3.9

**Table 9:** Dependence of changes in the density and workability of concrete mixtures on fiber content.

Composition	Density (kg/m^3^)	Slump (cm)
CC	2235	3.8
BF0.5	2241	3.7
BF1.0	2233	3.5
BF1.5	2237	3.2
BF2.0	2242	2.6
BF2.5	2238	2.1
BF3.0	2234	1.7
PF0.5	2235	3.7
PF1.0	2240	3.6
PF1.5	2238	3.0
PF2.0	2246	2.7
PF2.5	2241	2.0
PF3.0	2239	1.4

**Table 10:** Dependence of changes in the density and workability of concrete mixtures on microsilica content.

Composition	Density (kg/m^3^)	Slump (cm)
MS2	2237	3.6
MS4	2234	3.1
MS6	2241	2.7
MS8	2243	2.5
MS10	2245	2
MS12	2240	1.7

**Table 11:** Results of determining VCC density.

Composition	Density (kg/m^3^)	Δ (%)
CC	2398	0
BF0.5	2389	–0.38
BF1.0	2407	0.38
BF1.5	2422	1.00
BF2.0	2409	0.46
BF2.5	2417	0.79
BF3.0	2419	0.88
PF0.5	2397	–0.04
PF1.0	2409	0.46
PF1.5	2401	0.13
PF2.0	2398	0.00
PF2.5	2387	–0.46
PF3.0	2413	0.63
MS2	2402	0.17
MS4	2398	0.00
MS6	2411	0.54
MS8	2402	0.17
MS10	2385	–0.54
MS12	2379	–0.79

**Table 12:** Dependence of changes in the density and workability of concrete mixtures on the proportions of PF and BF.

Composition	Density (kg/m^3^)	Slump (cm)
BF1.5/PF0/MS8	2240	2.7
BF1.25/PF0.25/MS8	2239	2.5
BF1.0/PF0.5/MS8	2241	2.6
BF1.0/PF0.25/MS8	2245	2.7
BF0.75/PF0.75/MS8	2239	2.4
BF0.75/PF0.5/MS8	2342	2.9
BF0.75/PF0.25/MS8	2238	3.0

## Data Availability

Data are contained within the article.
